# Functionalization of gelatin films dressings with silver-doped zinc oxide nanoparticles for prevention of bacterial infection and promoting wound repair

**DOI:** 10.1038/s41598-025-13382-9

**Published:** 2025-08-12

**Authors:** Bahaa A. Hemdan, Samar A. El-Kholy, E. S. Shalaby, A. H. Abd-Al-Aleem, Tarek S. Aysha, Mehrez E. El-Naggar

**Affiliations:** 1 Water Pollution Research Department, Environment and Climate Change Research Institute, National Research Centre, 33 El-Bohouth St., Dokki, Giza 12622 Egypt; 2 Chemistry Department, Faculty of Science, Menoufia University, Shebin El Koom, 32511 Egypt; 3 Dyeing, Printing and Textile Auxiliaries Department, Textile Research and Technology Institute, National Research Centre, 33 El-Bohouth St., Dokki, Giza 12622 Egypt; 4 Pre-Treatment and Finishing of Cellulosic Fabric Department, Textile Research and Technology Institute, National Research Centre, 33 El-Bohouth St., Dokki, Giza 12622 Egypt

**Keywords:** Gelatin films, Silver-doped zinc oxide nanoparticles, Antibacterial activity, Biofilm inhibition, Biocompatibility, Wound healing

## Abstract

Gelatin-based films loaded with silver-doped zinc oxide nanoparticles (Ag-doped ZnONPs) were synthesized using the solution casting technique. The analyzed data of Ag-doped ZnONPs reveal their spherical shape with a small size. In our work, three different concentrations of the prepared Ag-doped ZnONPs (0.05 g, 0.1 g, and 0.2 g) were added to GeL solution for the formation of three films (Ag/ZnO 0.05@GeL film, Ag/ZnO 0.1@GeL film, and Ag/ZnO 0.2@GeL). These prepared films were compared with GeL film that prepared without Ag-ZnONPs. The obtained results demonstrated the relatively homogeneous distribution of Ag-ZnONPs on the surface of the prepared films. Besides, this study investigated the antibacterial properties, biocompatibility, and potential applications of Ag/ZnO 0.2@GeL film for wound healing. Ag/ZnO 0.2@GeL film exhibited the highest antibacterial efficacy, with zones of inhibition ranging from 17 to 21 mm against Gram-negative bacteria. Furthermore, this formulation showed a marked ability to inhibit biofilm formation, completely eradicating bacterial biofilm by day 7. In bacterial growth inhibition assays, Ag/ZnO 0.2@GeL reduced bacterial counts by up to 6 log CFU/mL within 210 min. Biocompatibility was assessed using a Microtox® analyzer, with EC_50%_ values exceeding 100 across all time points, confirming the films’ non-toxic nature. These findings suggest that Ag/ZnO@GeL films, particularly Ag/ZnO 0.2@GeL formulation, offer strong antibacterial activity, effective biofilm suppression, and high biocompatibility, highlighting their potential as multifunctional wound dressings for infection control and enhanced wound healing.

## Introduction

Wound healing is a complex and dynamic biological process that involves hemostasis, inflammation, proliferation, and tissue remodeling to restore the integrity of damaged skin and underlying tissues^1^. Likewise, the healing of wounds consists of four phases (hemostasis, inflammation, proliferation, and remodeling)^2^. Efficient wound healing is essential for infection prevention, functional recovery, and scar reduction^3^. However, wound infection continues to be a major clinical concern, especially in chronic wounds that are prone to delayed healing and extended inflammation, such as diabetic ulcers, surgical wounds, and burn injuries^4^. These infections often occur owing to bacterial pathogens, which may lead to skin infections, and consequences include sepsis, heightened inflammation, and impaired tissue regeneration^5^. Moreover, bacterial infections in wounds are primarily caused by opportunistic pathogens, including Gram-positive bacteria (*Staphylococcus aureus*) and Gram-negative bacteria (*Pseudomonas aeruginosa*), which are commonly found in hospital settings and chronic wound environments^6^.

Significantly, these bacteria compromise the healing process and form biofilm on wound surfaces, which act as protective barriers, making the bacteria more resistant to both the immune response and antibiotic treatments^4^. Biofilm development triggers a significant obstacle to effective wound management, contributing to persistent infections and chronic inflammation^7^. These structured microbial communities adhere to wound surfaces and are encapsulated within a self-produced extracellular polymeric substance (EPS), which protects bacteria from immune responses and antibiotic treatments^8^. Controlling the spread of pathogenic bacteria is essential for wound healing^9^. Conventional treatment strategies involve antibiotics, antiseptics, and sterile dressings. This highlights the urgent need for innovative and sustainable approaches to prevent and control bacterial infections in wounds^10^. One promising strategy is the use of antibacterial wound dressings that can not only protect the wound from disease but also promote healing. These dressings often incorporate materials with inherent antibacterial properties^11^.

Nanotechnology has emerged as a transformative tool across various biomedical fields, including drug delivery, tissue engineering, and wound care, due to the unique physicochemical and biological properties of nanomaterials^12^. Nanomaterials reveal essential physical, chemical, and biological properties absent in their bulk alternatives composed of the same substance^13^. Nano-based materials have emerged as a focal point of research due to their remarkable benefits, including bio-imaging capabilities, biocompatibility, ease of functionalization, and targeted tumor treatment^14^. Among several nanomaterials, zinc oxide nanoparticles (ZnONPs) have shown great promise in biological and biomedical applications because of their remarkable potential due to their intrinsic antibacterial activity, low toxicity, biocompatibility, and ability to generate reactive oxygen species (ROS) that selectively target bacterial cells without harming healthy tissues^15–17^. ZnONPs can interrupt biofilm development, prevent unchecked bacterial multiplication, and cause bacteria to undergo programmed cell death^18^. Crucially, they have no impact on healthy cells, which makes them a viable option for cancer treatment^19–21^.

The antibacterial efficacy of ZnONPs can be further enhanced by doping with silver nanoparticles (AgNPs), which boosts ROS production, increases membrane disruption, and intensifies oxidative stress within bacterial cells^22,23^. Combining AgNPs with ZnONPs increases oxidative stress and cell death in bacteria by boosting ROS generation inside bacterial cells^24^. This synergistic effect significantly enhances the antibacterial potential of ZnONPs, making them more effective in combating bacterial infections and potentially improving cancer treatments^25^. On the other hand, blending nanoparticles with natural polymers creates a composite combining both materials’ benefits. The nanomaterial (Ag-doped ZnONPs) is dispersed throughout the gelatin chains, playing as a nanofiller, and strengthening the unique characteristics such as (high surface-to-volume ratio and surface activity)^26^.

Gelatin (GeL), a biomacromolecule derived from collagen, has attracted considerable interest in biomedical applications due to its biocompatibility, biodegradability, low cost, film-forming ability, and structural similarity to native extracellular matrix^27,28^. These features make it an appealing platform for researching and developing advanced biomaterials in drug delivery and tissue engineering applications^29^. Nonetheless, GeL film has many drawbacks, including inadequate mechanical strength, reduced thermal stability, and inadequate barrier characteristics. The integration of biopolymers with various nanomaterials has shown effective in enhancing their useful properties and mitigating inherent limitations^30^. The incorporation of nanomaterials into biopolymer matrices can improve structural integrity, barrier properties, and biological activity, making these hybrid materials more suitable for demanding biomedical applications such as wound healing, drug delivery, and tissue regeneration^31,32^. In this respect, the current study developed the GeL-based films by adding glycerol as a plasticizing agent to modify their functional activities by manipulating their extensibility, dispensability, flexibility, elasticity, rigidity, and mechanical properties^33,34^. Furthermore, incorporating nanoparticles further enhances their physicochemical, thermal, mechanical, and biological properties of GeL films.

As a result of increasing antibiotic resistance, alternative antimicrobial wound dressings with enhanced properties need to be established, regardless of whether typical bandages remain beneficial. There is an increasing demand for developing complex wound dressings that protect the wound site, actively combat infection, and encourage the new tissue growth^35^. Despite the effectiveness of conventional dressings, the alarming rise in antibiotic resistance necessitates the development of alternative antibacterial strategies with enhanced properties. Nanoparticle-based wound dressings have emerged as a promising solution, offering potent antibacterial activity, biofilm inhibition, and improved healing outcomes^36^. While Ag and ZnONPs have individually demonstrated antimicrobial efficacy, their synergistic integration into a single nanocomposite structure (Ag-doped ZnONPs) offers enhanced functionality by combining ROS generation and membrane disruption mechanisms. This study presents a new formulation in which Ag-doped ZnONPs is embedded within a biocompatible and biodegradable gelatin (GeL) matrix to fabricate multifunctional wound dressing films. Our work presents three various concentrations of Ag-doped ZnONPs meticulously. The current research evaluates their physical and chemical characteristics, performance in bacterial eradication, ability to inhibit biofilm formation, capacity to safeguard cells from injury, and biocompatibility with cellular structures. The primary objective of this research is to evaluate the efficacy and biosafety of the enhanced Ag/ZnO@GeL formulation as an antibacterial wound dressing for addressing clinical issues related to multidrug-resistant pathogens and chronic wound infections associated with biofilm.

## Materials and methods

### Materials

Zinc chloride and silver nitrate were purchased from Sigma Aldrich Co., USA. Ammonia solution was purchased from El Nasr Pharmaceutical Chemicals Co., Egypt. Gelatin (GeL) and glycerol were purchased from Across Co. (Germany). All chemicals were used as received.

### Preparation of Ag-doped ZnONPs

Ag-doped ZnONPs was prepared according to the study reported by T. Siva Vijayakumar et al.^37^. For preparation, 0.2 M of zinc chloride (ZnCl_2_) and 0.001 M of silver nitrate (AgNO_3_) were blended, and mixed with 50 mL of distilled water. Then, ammonia solution (2 mL, 25%) was added dropwise until the formation of precipitation, followed by 10 drops, when the solution became transparent, exhibiting a silvery-white hue. The resultant solution was centrifuged for 60 min at 10000 rpm. The precipitate was collected and rinsed with deionized water and acetone, then centrifuged at 10000 rpm. The obtained precipitate (Ag-doped ZnONPs) was dried at 120 °C for 60 min.

### Preparation of gelatin films loaded with different concentrations of Ag-doped ZnONPs

2.5 g of GeL was dissolved in 100 mL of H_2_O with stirring at 60 °C until complete dissolution. For each 25 mL of GeL solution, 0.05, 0.1, and 0.2 g of Ag-doped ZnONPs were added with constant stirring at 60 °C for 60 min. Then, 2 mL of glycerol was added to the film solutions with stirring for 5 min at room temperature with continuous stirring. Finally, each solution was sonicated until the air bubbles were removed. The solutions coded as Ag/ZnO 0.05@GeL film, Ag/ZnO 0.1@GeL film, and Ag/ZnO 0.2@GeL film were cast in a mold and left for drying in air for 72 h. The formed dried films were stored into a desiccator until characterization and application. The films loaded with nanoparticles (Ag-doped ZnONPs) were compared to the Gel film without nanoparticles (GeL film).

### Characterization of Ag-doped ZnONPs and gelatin films

The shape and size distribution of the prepared Ag-doped ZnONPs were examined by Transmission electron microscopy (TEM, JEOL-2100, Japan). In addition, the selected area diffraction (SAD) was also assessed. The surface morphological features of the films (GeL film, Ag/ZnO 0.05@GeL, Ag/ZnO 0.1@GeL, and Ag/ZnO 0.2@GeL) were investigated using scanning electron microscope (SEM, Quartto S, Thermofisher, USA). FTIR coupled with platinum Diamond ATR (Bruker VERTEX 80, Germany) was used for the assessment of the functional groups of GeL film, Ag/ZnO 0.05@GeLfilm, Ag/ZnO 0.1@GeL film, and Ag/ZnO 0.2@GeL at a range of 4000–400 cm^−1^. The XRD patterns for the formed films were characterized using a Bruker D8 Advance at 40 kV. The spectra were obtained by applying Cu Kα radiation (1.54060) at 40 mA. The thermal characteristics of GeL film, Ag/ZnO 0.05@GeLfilm, Ag/ZnO 0.1@GeL film, and Ag/ZnO 0.2@GeL were evaluated by thermogravimetric analyzer using Perkin Elmer instrument (STA 8000 and 8500). TGA thermograms of the films were obtained by heating the samples from 50 to 600 °C at a rate of 10 °C/min in a nitrogen atmosphere. The water contact angle of the prepared films in terms of hydrophilicity/hydrophobicity nature was determined using a contact angle analyzer (OCA40, Dataphysics, Germany).

### In vitro antibacterial activity and culture conditions

The bacterial strains used in this study included *Pseudomonas aeruginosa*, *Acinetobacter baumannii*, *Staphylococcus epidermidis*, and *Streptococcus pyogenes*. These species were selected for their clinical relevance, particularly in healthcare-associated infections and wound-related complications. The antibacterial activity of four polymer-based film formulations (GeL film, Ag/ZnO 0.05@GeL film, Ag/ZnO 0.1@GeL film, and Ag/ZnO 0.2@GeL film), was evaluated using the agar diffusion method. Bacterial suspensions of *A. baumannii*, *P. aeruginosa*, *S. epidermidis*, and *S. pyogenes* were adjusted to a turbidity equivalent to 0.5 McFarland standard (about 1.5 × 10^8^ CFU/mL) and uniformly inoculated onto Muller-Hinton agar plates with sterile cotton swabs. Disks of the test films (2 × 2 cm) were aseptically placed onto the agar surface. Plates were incubated aerobically at 37 °C for 18–24 h. Zones of inhibition (ZOI) around the film disks were measured in mm^38^.

#### Bacterial growth inhibition assays

The antibacterial efficacy of the films was then evaluated by bacterial growth inhibition experiments. Film samples were introduced into sterile tubes containing new bacterial solutions. The tubes were incubated at 37 °C with continuous agitation to guarantee uniform dispersion. Bacterial proliferation was observed at time intervals from 0 to 210 min to assess time-dependent inhibition. At specified time intervals, aliquots were extracted, diluted with sterile saline, and inoculated into nutrient agar plates. Plates were incubated for 18 to 24 h to ascertain viable bacterial numbers^39^.

#### Protein leakage assay

The protein leakage assay was conducted to evaluate the effect of the films on bacterial membrane integrity. Bacterial suspensions were prepared in PBS at approximately 1.5 × 10^8^ CFU/mL. Under sterile conditions, the suspensions were exposed to the tested films (GeL film, Ag/ZnO 0.05@GeL film, Ag/ZnO 0.1@GeL film, and Ag/ZnO 0.2@GeL film). At predetermined time intervals (3, 6, and 12 h), aliquots were collected and centrifuged at 10000 × g for 10 min, and the supernatants were retained. Protein concentrations in the supernatants were measured using the Bradford assay^40^.

#### Biofilm reduction assays

The ability of the films to inhibit biofilm formation was tested using an in vitro biofilm model. Fresh bacterial suspensions (1.5 × 10^8^ CFU/mL) were prepared, and small film disks (2 × 2 cm) were aseptically placed in 24-well polystyrene plates containing 10 mL of inoculated medium per well. Wells without films served as controls to establish baseline biofilm formation. Incubation periods of 1, 3, 5, and 7 days were used to assess biofilm development. Bacterial adhesion and biofilm accumulation on the films were analyzed by scraping the biofilm from the film surfaces for further evaluation^41,42^.

### Measurements of intracellular reactive oxygen species (ROS)

The intracellular generation of ROS in bacterial cells treated with Ag/ZnO@GeL films was assessed using the fluorescent probe 2′,7′-dichlorodihydrofluorescein diacetate (DCFH-DA). Mid-log phase cultures of *P. aeruginosa*, *A. baumannii*, *S. epidermidis*, and *S. pyogenes* were harvested, washed with phosphate-buffered saline (PBS), and adjusted to an optical density of 0.5 at 600 nm. The bacterial cells were then incubated with 10 µM DCFH-DA at 37 °C in the dark for 30 min to allow probe uptake and intracellular deacetylation. After washing to remove excess dye, the bacteria were exposed to GeL film, Ag/ZnO 0.05@GeL film, Ag/ZnO 0.1@GeL film, and Ag/ZnO 0.2@GeL film. A group treated with 100 µM of hydrogen peroxide (H₂O₂) served as a positive control. The samples were incubated at 37 °C for 1 h, after which fluorescence intensity was measured at 485 nm excitation and 530 nm emission using a microplate reader^43^. ROS levels were expressed in relative fluorescence units (RFU) and normalized to bacterial density.

### Antioxidant assay using DPPH method

The antioxidant activity of the film formulations (GeL film, Ag/ZnO 0.05@GeL film, Ag/ZnO 0.1@GeL film, and Ag/ZnO 0.2@GeL film) was evaluated using the DPPH (2,2-diphenyl-1-picrylhydrazyl) assay. Film samples (2 × 2 cm) were incubated with 2 mL of 0.1 mM DPPH solution prepared in methanol^44^.

### Toxicological assessment using Microtox® analyzer

The toxicological profiles of GeL film, Ag/ZnO 0.05@GeL film, Ag/ZnO 0.1@GeL film, and Ag/ZnO 0.2@GeL film were evaluated using a Microtox® Model 500 analyzer. The analysis employed *Vibrio fischeri*, a bioluminescent bacterium, as the indicator organism. The films were pre-soaked in distilled water for five days, and the toxicity of the soaking solution was analyzed.

### Statistics

All experimental data were analyzed using GraphPad Prism software (version 8.0.1; GraphPad Software, Inc., USA). Results are expressed as mean ± standard deviation (SD) derived from at least three separate replicates. Statistical disparities across groups were evaluated using one-way analysis of variance (*ANOVA*).

## Results and discussion

### Characterization of Ag-doped ZnONPs

Initially, the synthesis of Ag-doped ZnONPs involved precipitating zinc chloride and silver nitrate with ammonia solution, forming Zn(OH)₂, AgOH, NH₄Cl, and NH₄NO₃. Sequential followed by washing with deionized water and acetone to form Zn(OH)₂ and AgOH. Thermal treatment at 120 °C produced Ag-doped-ZnONPs in a pure form. Then, Ag-doped ZnONPs with different concentrations were blended with GeL solution to fabricate a wound dressing film with enhanced efficacy against the pathogenic bacteria. Before film fabrication, the synthesized Ag-doped ZnONPs was characterized using TEM.

**Fig. 1:**
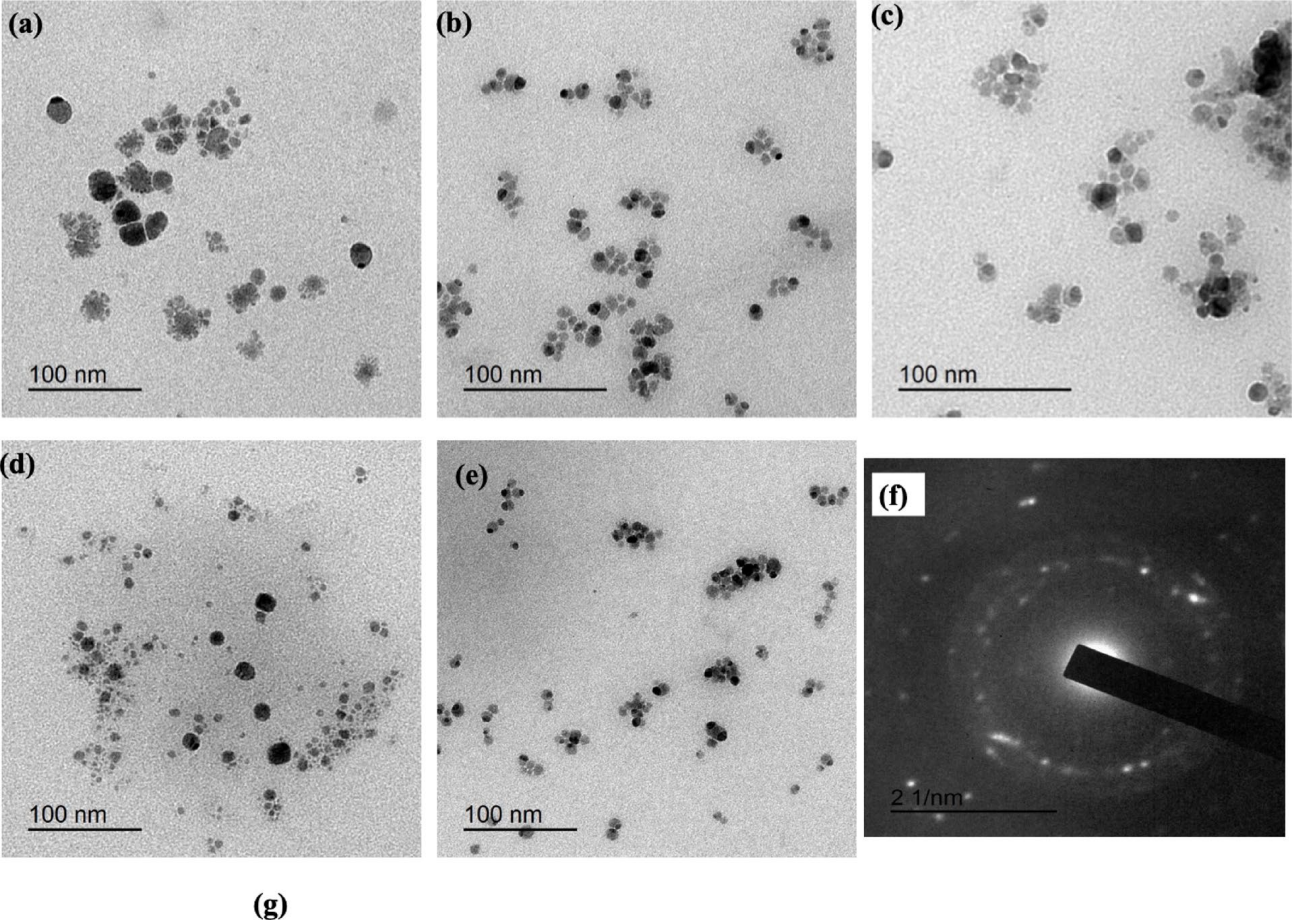
(**a**–**e**) TEM, and (**f**) SAD of Ag-doped ZnONPs.

The shape and size distribution of the obtained Ag-doped ZnONPs were assessed. Figure 1a–e reveals TEM images of Ag-doped ZnONPs using different positions. It was observed that the prepared Ag-doped ZnONPs was formed with small size and well distribution affirming the suitability of the co-precipitation technique for nanoparticles preparation. SAD of Ag-doped ZnONPs (Fig. 1f) shows discrete spots with irregular rearrangement indicating the polycrystalline nature of the formed Ag-doped ZnONPs. Also, some crystal planes were manifested, for instance, the (100), (102), (101), and (002) planes attributed to wurtzite structure of ZnONPs whereas the (005), (006), and (007) planes for AgNPs^45,46^.

### Characterization of Ag-doped ZnONPs loaded gelatin films

**Fig. 2:**
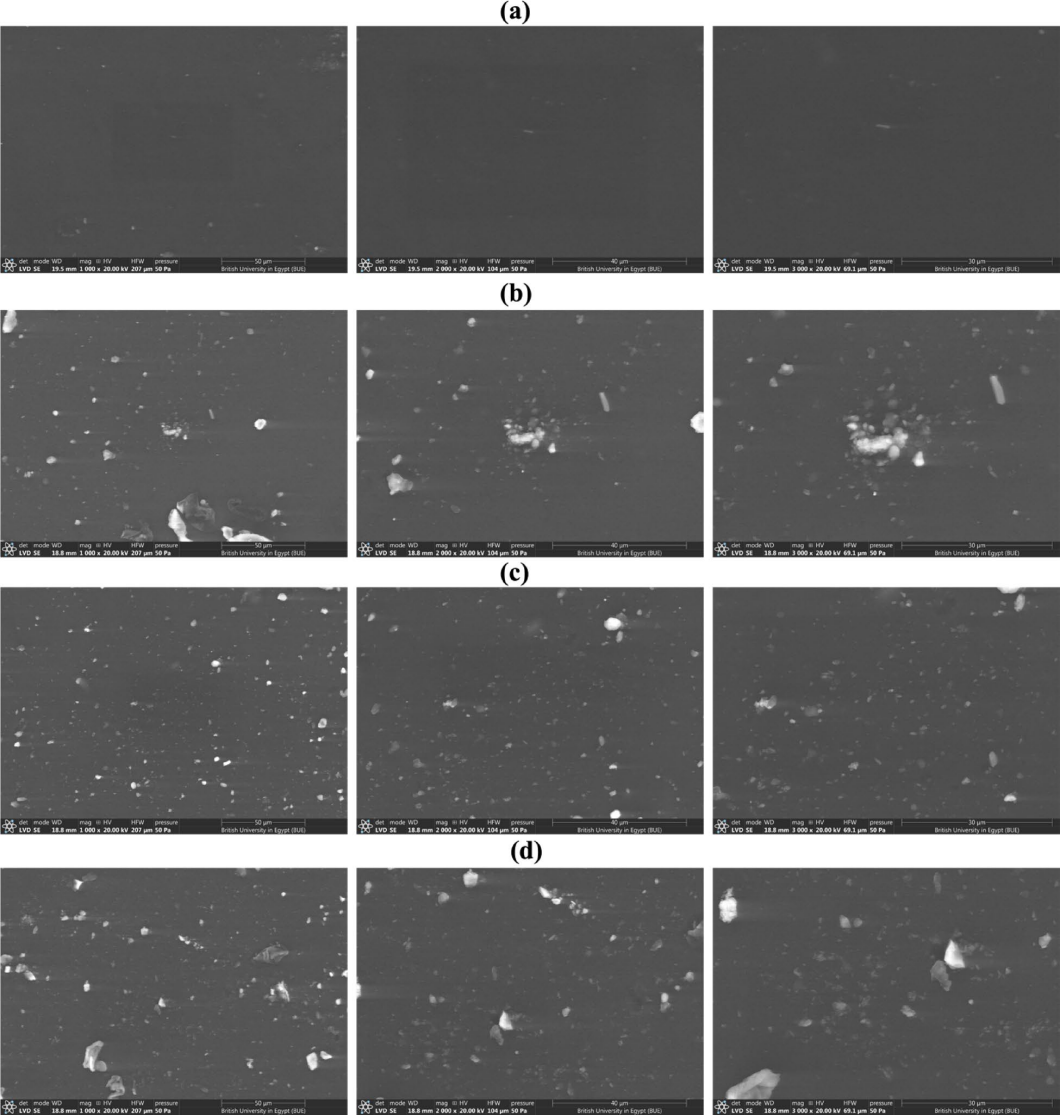
SEM images of (**a**) GeL film, (**b**), Ag/ZnO 0.05@ GeL film, (**c**) Ag/ZnO 0.1@ GeL film and (**d**) Ag/ZnO 0.2@GeL film at three different magnifications.

The morphological structure of GeL films in the absence and presence of Ag-doped ZnONPs were examined using SEM at three different magnifications. SEM images of GeL and Ag/ZnO@GeL films are illustrated in Fig. 2. SEM revealed a homogeneous and smooth surface topology for the GeL film (Fig. 2a). GeL films loaded with different concentrations of Ag-doped ZnONPs (Ag/ZnO 0.05@GeLfilm, Ag/ZnO 0.1@GeL film, and Ag/ZnO 0.2@GeL) exhibited different surface topologies as displayed in Fig. 2b–d, respectively. SEM analysis depicted the surface roughness due to the deposition of Ag-doped ZnONPs onto the surface of GeL films, confirming effective functionalization of GeL film with nanoparticles. The synthesized films (GeL film, Ag/ZnO 0.05@GeL film, Ag/ZnO 0.1@ GeL film, and Ag/ZnO 0.2@GeL film) demonstrated compact surfaces with high structural integrity, devoid of defects such as cracks or pores. SEM revealed a relatively uniform distribution of Ag-doped ZnONPs, and the deposited particle were increased proportionally with the added concentration of nanoparticles.

**Fig. 3:**
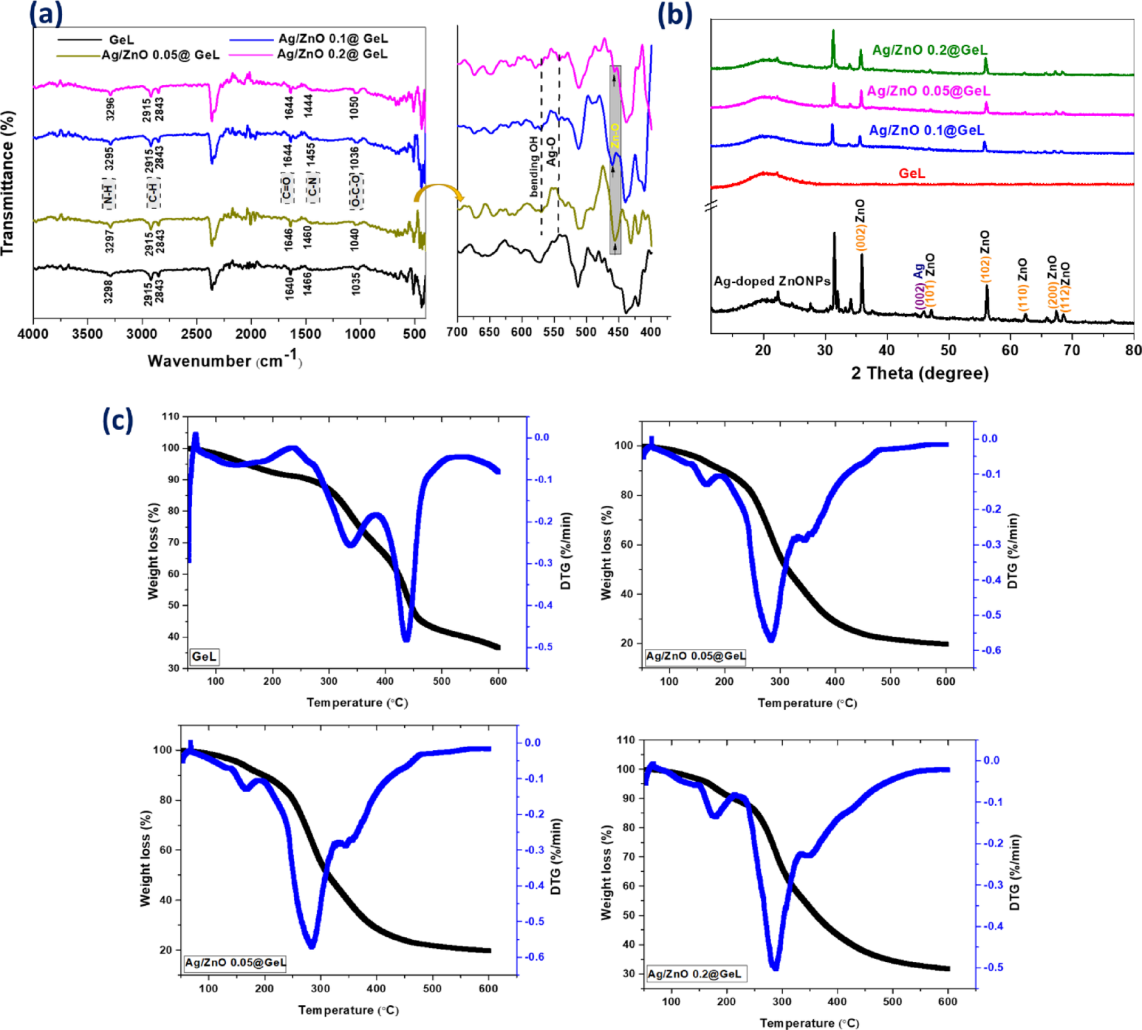
(**a**) FTIR, (**b**) XRD and (**c**) TGA and DTG of GeL film, Ag/ZnO 0.05@GeL film, Ag/ZnO 0.1@GeL film and (**d**) Ag/ZnO 0.2@GeL film.

FTIR spectra of pristine GeL film, and functionalized GeL films with different concentrations of Ag-doped ZnONPs were scanned in the range 4000–400 cm^−1^ as illustrated in Fig. 3a. The GeL film exhibited unique peaks, which confirmed its chemical structure. For instance, the band at 3298 cm^-1^ is related to the N–H stretching vibration of secondary amide. In contrast, the bands at 2915 and 2843 cm^−1^ corresponded to the asymmetric and symmetric stretching modes of saturated C–H groups. The carbonyl (C=O) stretching vibration of amide Ι was recorded at a band of 1640 cm^−1^, while the C–N stretching of amide ΙΙ and the O–C–O vibration are located at 1466 and 1035 cm^−1^, respectively. The bands located at 2358 and 2368 cm^−1^ may be attributed to the atmospheric CO_2_ present in the instrument^47,48^. For the functionalized GeL films with Ag-doped ZnONPs (Ag/ZnO 0.05@GeL film, Ag/ZnO 0.1@GeL film, and Ag/ZnO 0.2@GeL film), similar peaks were observed with minor shifts in their positions, verifying that Ag-doped ZnONPs bound with GeL chains and did not alter the chemical composition of GeL. For example, N–H shifted to lower wavenumbers (from 3298 to 3297, 3295, and 3296 cm^−1^ for Ag/ZnO 0.05@GeL film, Ag/ZnO 0.1@GeL film and Ag/ZnO 0.2@GeL film, respectively), also, the band of C–N was moved to 1460, 1455, and 1444 cm^−1^, respectively. Whereas, C=O and C–O were shifted to higher wavenumbers. For C=O, the peak position was changed from 1640 into 1646, 1644, and 1644 cm^−1^, respectively. Meanwhile, C–O was shifted from 1035 to 1040, 1036, and 1050 cm^−1^, respectively. Furthermore, some new peaks signified the successful incorporation of Ag-doped ZnONPs. Where, in the lower frequency range (700–400 cm^−1^), the band at 566 cm^−1^ is related to O–H (of some water molecules and Zn (OH)_2_ that formed as an intermediate during ZnONPs preparation). Further, bands at 542 and 455 cm^−1^ correspond to Ag–O and Zn–O, respectively. These peaks are bound with active parts of GeL film, changing their positions into lower wavenumbers, as mentioned above.

Figure 3b displayed the XRD spectra of GeL films without and with different concentrations of Ag-doped ZnONPs. The obtained data of Ag-doped ZnONPs exhibited peaks at 2θ = 35.9°, 46.1°, 47.2°, 56.18°, 62.5°, 67.59°, and 68.6° are related to (002), (101), (102), ((110), (200), and (112) of hexagonal wurtzite ZnONPs. Meanwhile, the diffraction (002) plane at 2θ = 45.92° is corresponds to the doped AgNPs. For GeL film, an intensified diffraction peak was recognized at 2θ = 19.9°, which reveals the semicrystalline nature of GeL due to the rearrangement of their chains in helix and triple-helical structures. After incorporating different concentrations of Ag-doped ZnONPs, the crystallinity of Gel films is enhanced, and the distinguished peaks of Ag-doped ZnONPs were observed. The 002 diffraction peak of GeL became less intense, broader, and shifted into 20.2°, 20.5°, and 20.9° for Ag/ZnO 0.05@GeL, Ag/ZnO 0.1@GeL, and Ag/ZnO 0.2@GeL, respectively. Also, (002), (102), (200), and (112) of ZnONPs appeared in the functionalized GeL films, and its intensity increased with increasing the concentration of Ag-doped ZnONPs.

TGA, % and its first derivative (DTG, %/min) of GeL film, Ag/ZnO 0.05@GeL film, Ag/ZnO 0.1@GeL film, and Ag/ZnO 0.2@GeL film were presented in Fig. 3c. GeL films have multi-decomposition stages related to water evaporation, glycerol and GeL polymer degradation. The moisture removal, from unfunctionalized and functionalized GeL films with Ag-doped ZnONPs, was observed around 100 ºC with weight loss in the range of 6–9.47%. whereas, glycerol degradation was monitored as a small shoulder at around 210–260 °C^49^. Additionally, the remained stages were related to polymer decomposition. For GeL film, the GeL degradation was detected at 270–550 °C with a weight loss of 55.32%. A significant reduction in the weight loss was observed after the incorporation of Ag-doped ZnONPs. Because the added Ag-doped ZnONPs comprised metal and metal oxide, which are inorganic, and exhibited high thermal stability compared to the Gel film, therefore, the output thermal stability of the functionalized GeL films was enhanced. The degradation of Ag/ZnO 0.05@GeL film was observed at 275–500 °C, with a weight loss of 42.1%. While, Ag/ZnO 0.1@GeL film was detected at 280–515 °C with weight loss 41.99%, and Ag/ZnO 0.2@GeL film was observed at 396–511 °C with weight loss reached 29.18%, respectively. It was observed that the addition of Ag-doped ZnONPs enhanced the thermal stability of GeL films. Ag/ZnO 0.2@GeL film showed higher observable thermal stability rather than other functionalized (Ag/ZnO 0.05@GeL film and Ag/ZnO 0.1@GeL film) and pristine GeL film. Subsequently, the Ag-doped ZnONPs served as thermal insulator or a mass transport barrier to the volatile products generated during thermal decomposition^50,51^.

**Fig. 4:**
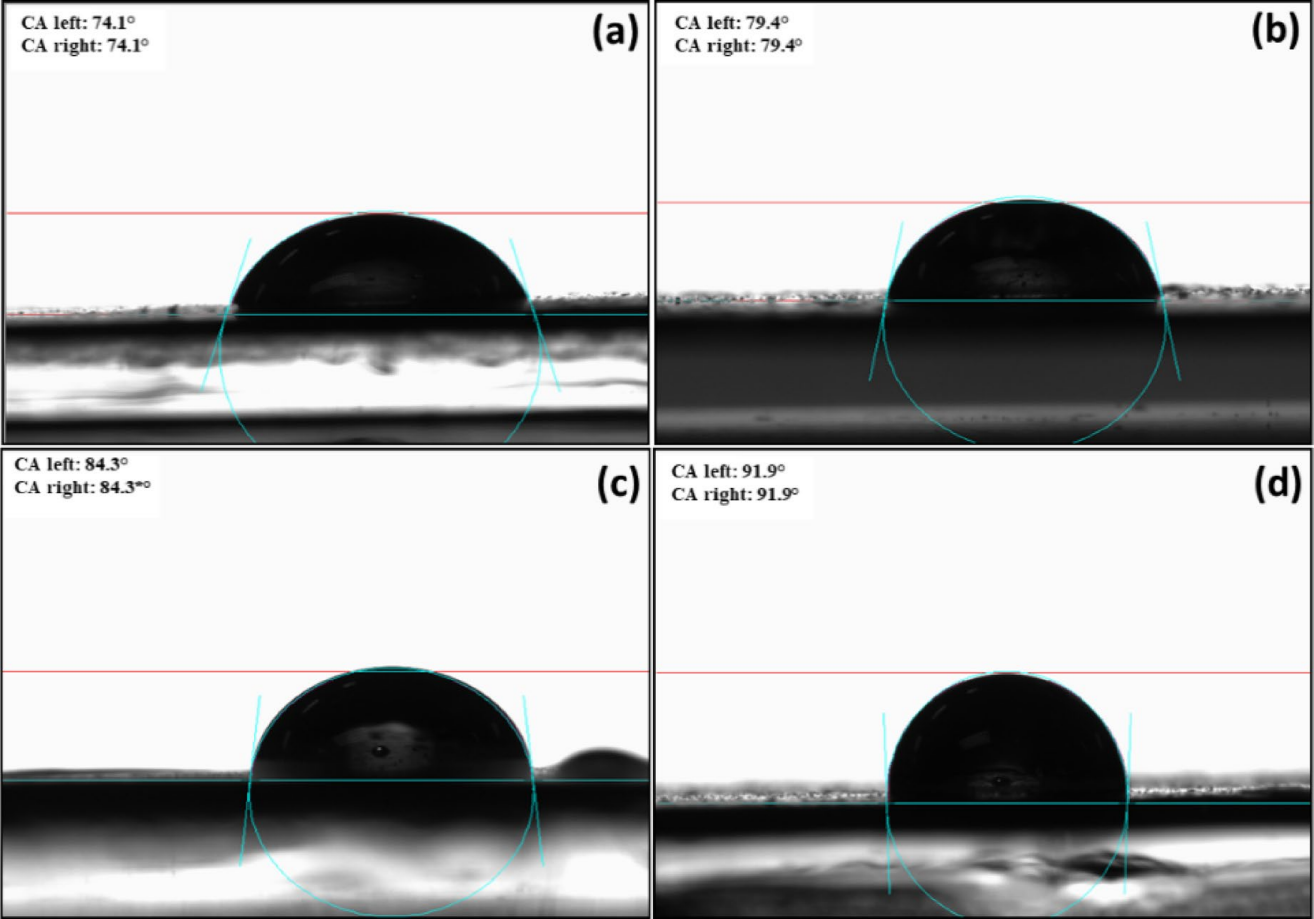
Water contact angle of (**a**) GeL film, (**b**) Ag/ZnO 0.05@GeL film, (**c**) Ag/ZnO 0.1@GeL film and (**d**) Ag/ZnO 0.2@GeL film.

The wettability and surface hydrophobicity/ hydrophobicity) of GeL film, Ag/ZnO 0.05@GeL film, Ag/ZnO 0.1@GeL film and Ag/ZnO 0.2@GeL film were evaluated by assessing the contact angle of the water droplets on the surface of the films^52^. Water contact angle (WCA) is an important indicator of surface hydrophobicity, and its value is associated with microstructure, chemical features, crystallinity, and roughness^53,54^. As known, a higher WCA value (θ > 90°) indicate hydrophobic surface while a smaller WCA (θ < 90°) refers the hydrophilic properties of the materials. WCA of pristine and functionalized GeL films were displayed in Fig. 4. The measured WCA of GeL film was 74.1° (Fig. 4a) reflecting the hydrophilic nature of GeL due to hydrophilic groups such as O–H, C=O, and N–H groups that are easily attracted to the water molecules.

Incorporation of Ag-doped ZnONPs with GeL films improved the surface hydrophobicity, where, the WCA values were changed from 74.1° (GeL film) to 79.4° (Fig. 4b), 84.3° (Fig. 4c) ,and 91.9° (Fig. 4d) for Ag/ZnO 0.05@GeL film, Ag/ZnO 0.1@GeL film, and Ag/ZnO 0.2@GeL film, respectively. That is due to (1) formation of further hydrogen bonds among GeL chains causing a decrease in the free hydrophilic groups. Also, (2) the incorporated Ag-doped ZnONPs altered the microstructure of GeL film and thus, enhancing the film crystallinity.

### Evaluation of antibacterial activities of films

#### Measuring of zone of inhibition (ZOI)

**Fig. 5:**
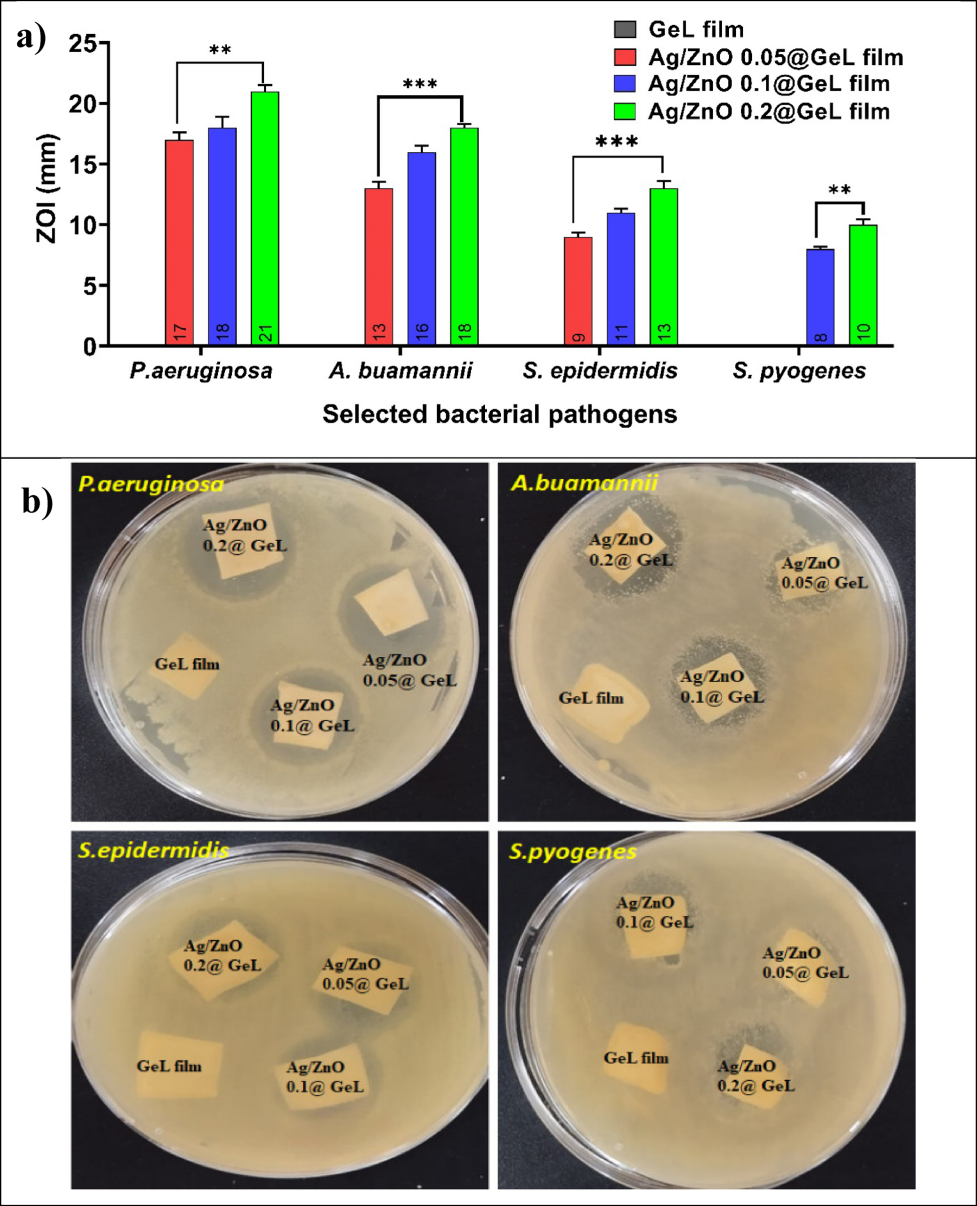
(**a**) the recorded values ZOI of GeL film, Ag/ZnO 0.05@GeL film, Ag/ZnO 0.1@GeL film, and Ag/ZnO 0.2@GeL film against *P. aeruginosa*, *A. baumannii*, *S. epidermidis*, and *S. pyogenes*. The values represent the mean ZOI (mm) with statistical significance indicated (**p < 0.01, ***p < 0.001), (**b**) the real photographic images of ZOI observed in agar plates.

Various studies have demonstrated that Ag-doped ZnONPs inhibit bacterial growth and promote fibroblast proliferation and migration, which are critical for wound healing^55^. Thus, the GeL film was incorporated with Ag-doped ZnONPs. Figure 5a,b indicates the ZOI results that reveal the antibacterial activity of the film formulations (GeL film, Ag/ZnO 0.05@GeL film, Ag/ZnO 0.1@GeL film, and Ag/ZnO 0.2@GeL film) against all tested bacterial strains, including *P. aeruginosa*, *A. baumannii*, *S. epidermidis*, and *S. pyogenes*. For *P. aeruginosa*, the ZOI values were 17 mm for Ag/ZnO 0.05@GeL film, 18 mm for Ag/ZnO 0.1@GeL film, and 21 mm for Ag/ZnO 0.2@GeL film, demonstrating a significant increase with higher Ag-doped ZnONPs concentrations. Similarly, for *A. baumannii*, ZOI values were 13 mm, 16 mm, and 18 mm for Ag/ZnO 0.05@GeL film, Ag/ZnO 0.1@GeL film, and Ag/ZnO 0.2@GeL film, respectively. In the case of *S. epidermidis*, ZOI values ranged from 9 mm for Ag/ZnO 0.05@GeL film to 11 mm for Ag/ZnO 0.1@GeL film and 13 mm for Ag/ZnO 0.2@GeL film. Lastly, for *S. pyogenes*, the ZOI values were 8 mm for Ag/ZnO 0.05@GeL film, 9 mm for Ag/ZnO 0.1@GeL film, and 10 mm for Ag/ZnO 0.2@GeL film (Fig. 5a). Further, the actual photographic images of ZOI in agar plates are illustrated in Fig. 5b. of These results demonstrate that Ag/ZnO 0.2@GeL film consistently achieved the highest ZOI for all tested pathogens. Notably, gram-negative bacteria (*P. aeruginosa* and *A. baumannii*) showed higher susceptibility (ZOI range: 13–21 mm) compared to gram-positive bacteria (*S. epidermidis* and *S. pyogenes*, ZOI range: 8–13 mm).

This data supports the superior antibacterial efficacy of Ag/ZnO 0.2@GeL film, attributed to the membrane-disrupting properties of Ag-doped ZnONPs and their ability to generate ROS, which interfere with bacterial viability. Doping ZnONPs with AgNPs enhances the generation of ROS when exposed to light, particularly visible light. This is attributed to the surface plasmon resonance of Ag, which improves light absorption and leads to an increased production of hydroxyl radicals (^·^*OH*) and superoxide anions (^·^*O*_2_^−^). These ROS have powerful antibacterial effects against prevalent bacterial infections by efficiently destroying bacterial membranes surrounding cells^56^. Additionally, the antibacterial activity and biological safety of the biosynthesized ZnONPs were evaluated against various pathogenic bacteria. The ZnONPs demonstrated measurable ZOI, with diameters of 12 mm for *E. coli*, 18 mm for *P. aeruginosa* and *K. pneumoniae*, and 20 mm for both *B. subtilis* and *S. aureus*^57^.

#### Inhibitory effect of selected bacterial pathogens

**Fig. 6:**
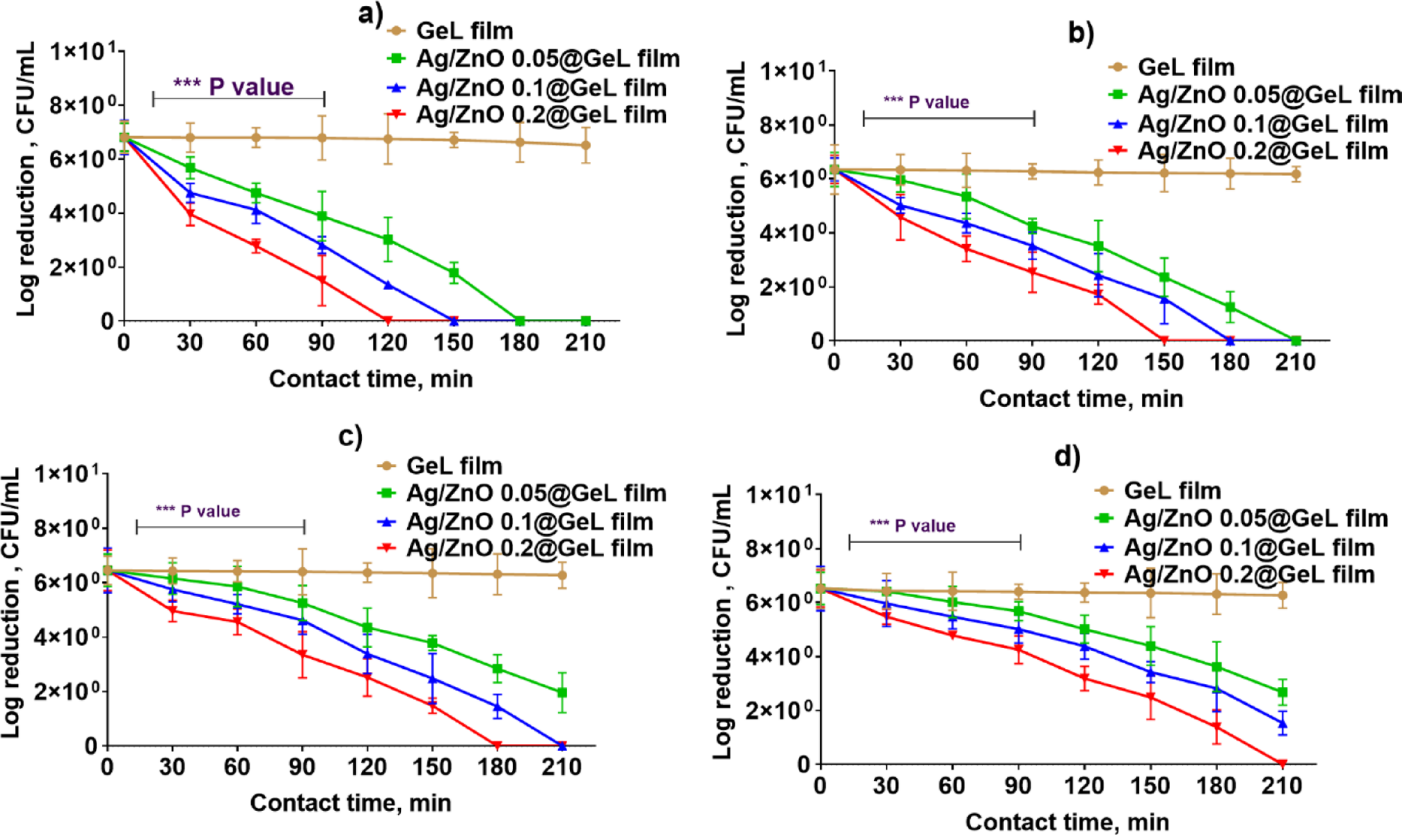
Killing kinetics of GeL film, Ag/ZnO 0.05@GeL film, Ag/ZnO 0.1@GeL film, and Ag/ZnO 0.2@GeL film against (**a**) *P. aeruginosa,* (**b**), *A. baumannii,* (**c**) *S. epidermidis*, and (**d**) *S. pyogenes*. The graphs show a log reduction in bacterial population (CFU/mL) over time, with statistical significance indicated (**p < 0.01, ***p < 0.001).

Figure 6 illustrate the bacterial growth inhibition and killing kinetics of the films over 210 min, showing the efficacy of different film formulations (GeL film, Ag/ZnO 0.05@GeL film, Ag/ZnO 0.1@GeL film, and Ag/ZnO 0.2@GeL film) against various bacterial strains, including *P. aeruginosa*, *A. baumannii*, *S. epidermidis*, and *S. pyogenes*. In Fig. 6a, the killing kinetics against *P. aeruginosa* demonstrate a substantial decreasing in bacterial counts with increasing concentrations of Ag-doped ZnONPs. The GeL film showed no reduction in bacterial populations, maintaining a relatively stable log count throughout the 210 min. The Ag/ZnO 0.2@GeL film formulation achieved complete bacterial eradication (6 log reduction) around 150 min, significantly outperforming the other formulations. The Ag/ZnO 0.1@GeL film showed substantial reduction, but complete eradication was not reached within 210 min. Ag/ZnO 0.05@GeL film displayed a moderate reduction in bacterial population, but full eradication was not observed by the end of the experiment. Statistical significance (*p* < 0.001) was observed for Ag/ZnONPs@GeL films compared to GeL film, highlighting the enhanced antibacterial properties of these formulations.

Figure 6b shows similar trends for *A. baumannii*, with GeL film showing little to no effect on bacterial growth. The Ag/ZnO 0.2@GeL film formulation again achieved the highest log reduction, demonstrating superior antibacterial activity. Ag/ZnO 0.1@GeL film and Ag/ZnO 0.05@GeL film exhibited moderate effects, gradually decreasing bacterial populations over time and confirming the films’ efficacy against this resistant pathogen. As shown in Fig. 6c, the data for *S. epidermidis* follows a similar pattern. The GeL film had negligible activity, while all films loaded with Ag-doped ZnONPs displayed improved antibacterial effects. Again, the Ag/ZnO 0.2@GeL film led to the most significant reduction in bacterial load, effectively reducing the population to 6 log CFU/mL by 210 min. This result underlines the enhanced antibacterial potential of Ag-doped ZnONPs that are incorporated in the film matrix.

Figure 6d represents the effect of the films on *S. pyogenes*. As with the other bacterial strains, GeL film showed little to no antibacterial activity, while Ag/ZnO 0.2@GeL film exhibited a significant log reduction in bacterial count over time. The Ag/ZnO 0.1@GeLfilm and Ag/ZnO 0.05@GeL film showed moderate bacterial suppression, though they were less effective compared to Ag/ZnO 0.2@GeL film.

The Ag/ZnO 0.2@GeL film consistently exhibited the most potent antibacterial activity, achieving complete bacterial eradication within the shortest time for all bacterial strains tested. These findings highlight the Ag/ZnO 0.2@GeL film as promising candidates for wound dressing applications, offering both rapid bacterial killing and the potential for enhanced wound healing through infection control. The lower concentration formulations (Ag/ZnO 0.1@GeL film and Ag/ZnO 0.05@GeL film) also demonstrated efficacy but required longer times to achieve substantial bacterial reduction.

The more potent antibacterial activity of Ag/ZnO@GeL films against Gram-negative bacteria can be attributed to the differences in cell wall structure, membrane permeability, and susceptibility to Ag⁺ and ROS. The outer membrane of Gram-negative bacteria provides less resistance to these antibacterial agents, allowing for more efficient penetration and more significant antibacterial effects. Conversely, Gram-positive bacteria possess a thicker cell wall and less permeable membrane^58^. The enhanced antibacterial activity of Ag-doped ZnONPs can be attributed to the synergistic effects of Ag-doped ZnONPs have been shown to generate ROS upon exposure to bacteria, which induces oxidative stress and causes damage to bacterial cell membranes^59^. ROS, including hydroxyl radicals and hydrogen peroxide, are highly reactive molecules that can oxidize lipids, proteins, and DNA within bacterial cells, leading to membrane disruption and cell death. In addition to the ROS production by ZnO, Ag ions further enhance ROS generation, intensifying oxidative stress within the bacterial cells. The Ag ions (Ag⁺) are known to interact with thiol groups in bacterial proteins, disrupting enzyme functions and compromising cell integrity^60^. Moreover, Ag⁺ ions can also interact with bacterial DNA, inhibiting replication and leading to cell death^61^. This synergistic process complicates bacterial resistance development, since the combined oxidative stress and disruption of cellular functioning across numerous pathways provide a more complex challenge for bacteria than single-target antibacterial drugs. This tackles a critical issue in antimicrobial resistance (AMR), whereby bacteria develop strategies to deactivate or eliminate antibacterial agents^62^.

#### Assessment of released protein

**Fig. 7:**
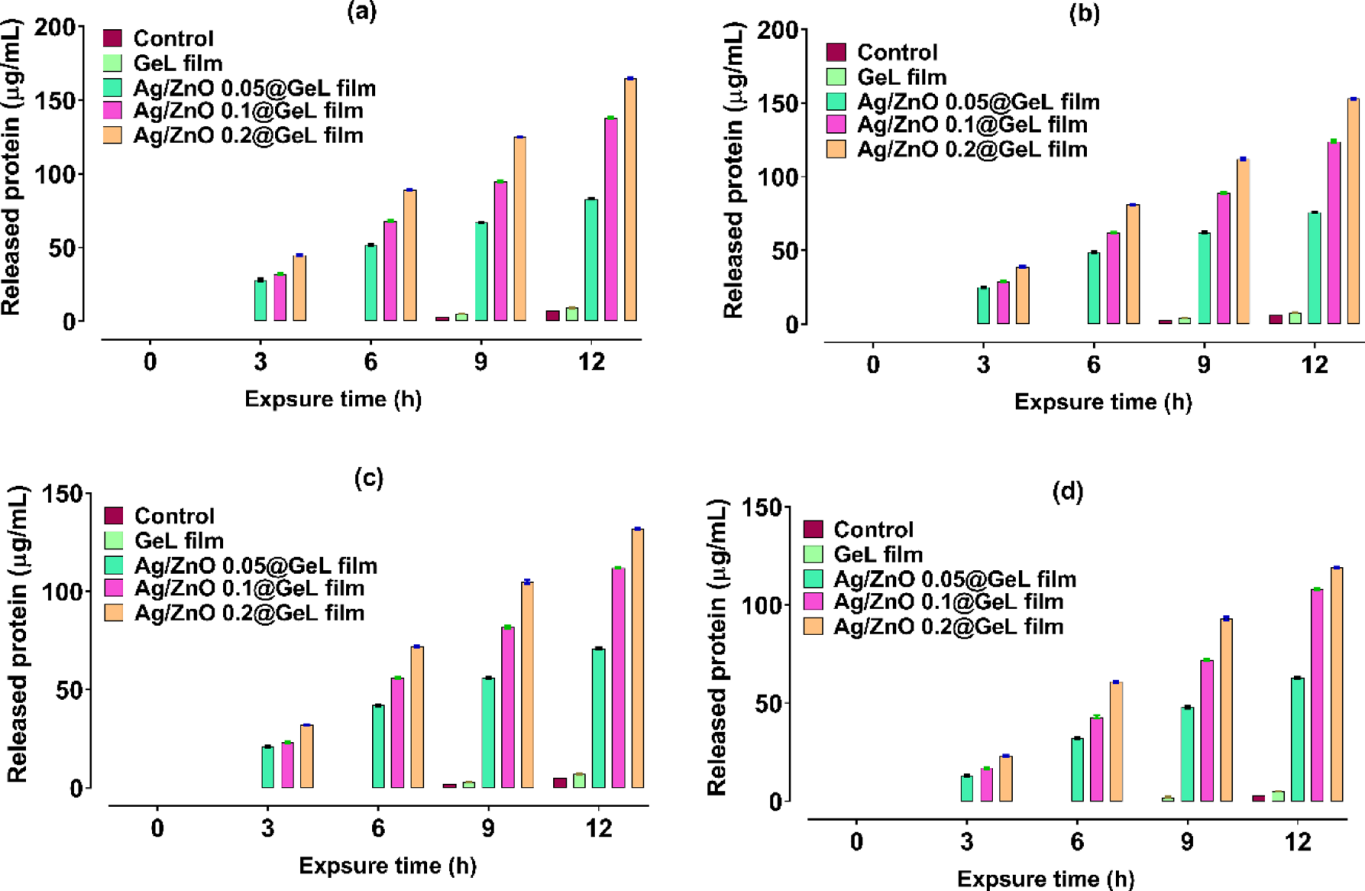
Protein release (µg/mL) at 3, 6, 9, and 12 h from damaged bacterial cells induced by different films, GeL film, Ag/ZnO 0.05@GeL film, Ag/ZnO 0.1@GeL film, and Ag/ZnO 0.2@GeL film, against *P. aeruginosa* (**a**), *A. baumannii* (**b**), *S. epidermidis* (**c**), and *S. pyogenes* (**d**).

Protein release assays were conducted to assess the degree of membrane damage and changes in cellular permeability induced by the different films (GeL film, Ag/ZnO 0.05@GeL film, Ag/ZnO 0.1@GeL film, and Ag/ZnO 0.2@GeL film). These films were tested against *P. aeruginosa*, *A. baumannii*, *S. epidermidis*, and *S. pyogenes* to evaluate their effects on bacterial cell integrity (Fig. 7). In Fig. 7a, the amount of released protein from *P. aeruginosa* bacterial cells was measured, the GeL film showed minimal protein release, with values of 28 µg/mL at 3 h, 32 µg/mL at 6 h, and 45 µg/mL at 12 h, indicating a low impact on bacterial membrane disruption. The Ag/ZnO 0.05@GeL film exhibited moderate protein release, with 52 µg/mL at 6 h and 89 µg/mL at 12 h. Increasing the concentration of Ag-doped ZnONPs resulted in higher protein release, with Ag/ZnO 0.2@GeL film reaching 125 µg/mL at 6 h and 165 µg/mL at 12 h, suggesting a stronger antibacterial effect.

In Fig. 7b, similar trends in *A. baumannii* were observed. The GeL film released 25 µg/mL of protein at 3 h and 39 µg/mL at 12 h, indicating minimal disruption of bacterial membranes. The Ag/ZnO 0.05@GeL film showed higher protein release, reaching 49 µg/mL at 6 h and 81 µg/mL at 12 h. Both Ag/ZnO 0.1@GeL film and Ag/ZnO 0.2@GeL film exhibited an increase in protein release over time, with Ag/ZnO 0.2@GeL film reaching 112 µg/mL at 9 h and 153 µg/mL at 12 h, indicating more extensive membrane disruption. The results illustrated in Fig. 7c have a similar pattern, with the GeL film causing low protein release in *S. epidermidis*, at 21 µg/mL at 3 h and 32 µg/mL at 12 h. The Ag/ZnO 0.05@GeL film released 56 µg/mL at 6 h and 72 µg/mL at 12 h. The Ag/ZnO 0.2@GeL film induced the highest protein release, with 105 µg/mL at 6 h and 132 µg/mL at 12 h, suggesting substantial membrane damage.

Finally, in Fig. 7d, the GeL film showed the lowest protein release from *S. pyogenes*, with 13 µg/mL at 3 h and 23 µg/mL at 12 h. The Ag/ZnO 0.05@GeL film resulted in a higher protein release of 32 µg/mL at 6 h and 61 µg/mL at 12 h. The Ag/ZnO 0.1@GeL film and Ag/ZnO 0.2@GeL film exhibited the most significant protein leakage, with 93 µg/mL at 9 h and 119 µg/mL at 12 h for the 0.2% formulation. In summary, the Ag/ZnO 0.2@GeL films consistently induced the highest levels of protein release across all bacterial strains, indicating significant membrane disruption. As the Ag-doped ZnONPs concentration increased, the degree of protein leakage increased, highlighting the enhanced antibacterial properties of the Ag/ZnO@GeL films. The GeL film, on the other hand, demonstrated minimal membrane damage, suggesting its limited antibacterial effect. These findings emphasize the potential of Ag/ZnO@GeL film for wound healing applications, where membrane disruption plays a crucial role in combating bacterial infections.

#### Exploring the control of biofilm formation

Biofilm is a complex, organized structure formed by microorganisms that adhere to surfaces and are embedded in a self-produced extracellular polymeric substance (EPS) matrix^63^. This biological layer protects the microbes from external threats, including antibiotics, immune responses, and other antibacterial agents. The formation of biofilm is a major concern in chronic infections, as bacteria within biofilm are significantly more resistant to treatments compared to planktonic (free-floating) bacteria^64^.

**Fig. 8:**
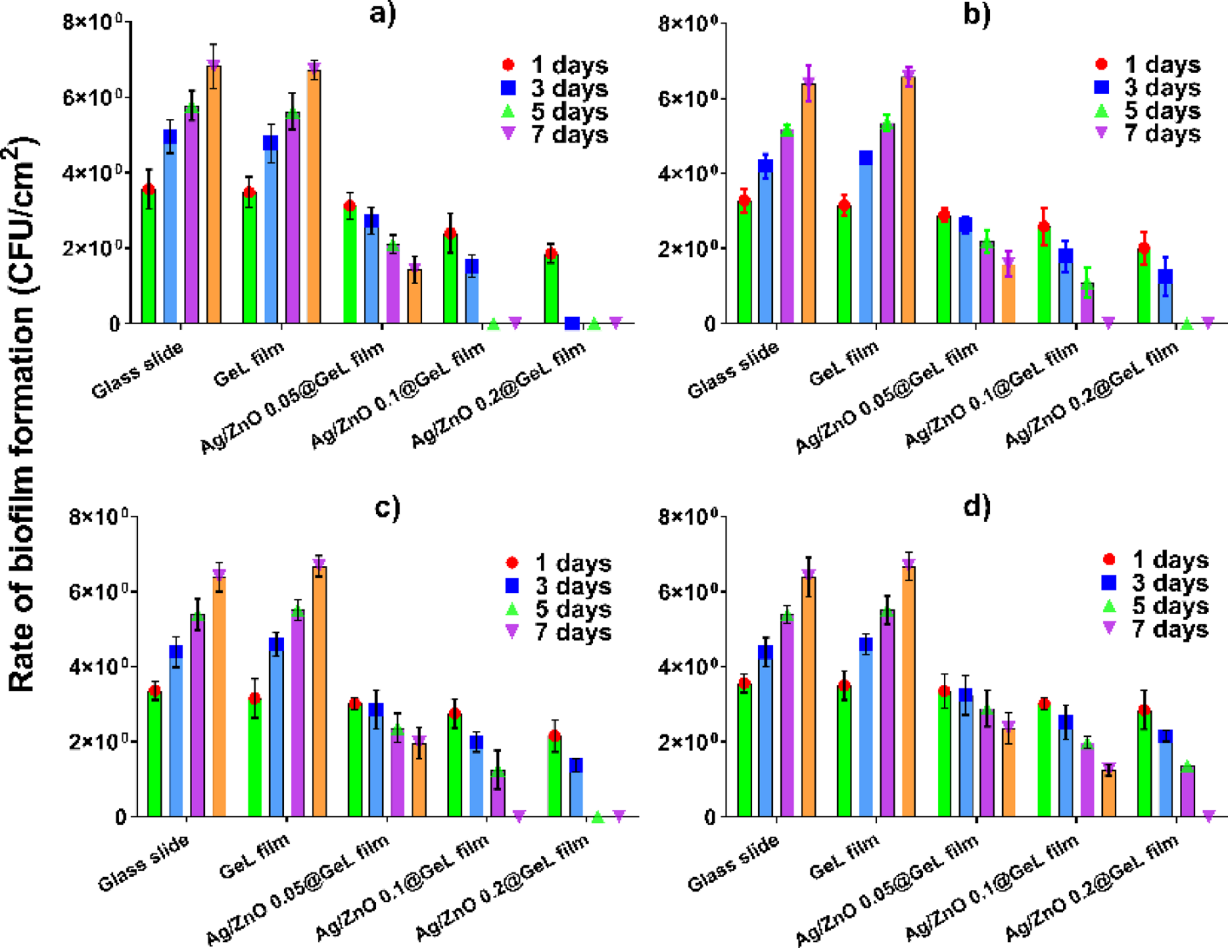
Rate of biofilm formation (CFU/cm^2^) of (**a**) *P. aeruginosa A. baumannii.* (**b**), (**c**) *S. epidermidis*, and (**d**) *S. pyogenes* on GeL film, Ag/ZnO 0.05@GeL film, Ag/ZnO 0.1@GeL film, and Ag/ZnO 0.2@GeL film after 1, 3, 5, and 7 days of incubation, compared to the control materials (glass slides and GeL film).

In this study, we investigated the ability of Ag/ZnO@GeL films to inhibit biofilm formation, focusing on their effectiveness against key wound-related pathogens. The results provide valuable insights into the potential of these films to combat biofilm-associated infections, which are common in chronic wound healing. Figure 8 demonstrates biofilm formation on the surface of various films (GeL film, Ag/ZnO 0.05@GeL film, Ag/ZnO 0.1@GeL film, and Ag/ZnO 0.2@GeL film) tested against *P. aeruginosa*, *A. baumannii*, *S. epidermidis*, and *S. pyogenes* over incubation time. The data provides insight into how these films impact bacterial adhesion and biofilm development, which is critical for wound healing applications. In Fig. 8a, the GeL film showed the highest biofilm formation of *P. aeruginosa* with a count of 5.8 at 1 day, which decreased to 0 by 7 days, indicating some level of inhibition over time. The Ag/ZnO 0.05@GeL film reduced biofilm formation to 4.8 at 1 day, further dropping to 0 by 7 days. Ag/ZnO 0.1@GeL film showed similar trends, starting at 3.5 at 1 day and reaching 0 at 7 days. The Ag/ZnO 0.2@GeL film exhibited the most significant reduction, with 6.7 at 1 day, decreasing to 0 by 7 days, suggesting a substantial impact on biofilm formation.

As represented in Fig. 8b, GeL film exhibited a notable reduction in *A. baumannii* biofilm formation, with the cell count decreasing from 3.3 on day 1 to 0 by day 7. The Ag/ZnO 0.05@GeL film also demonstrated biofilm inhibition, reducing the count from 4.2 on day 1 to 1.6 by day 7. The Ag/ZnO 0.1@GeL film formulation showed slightly greater efficacy, decreasing biofilm levels from 3.2 to 1.1 over the same period. The most pronounced effect was observed with the Ag/ZnO 0.2@GeL film, which reduced biofilm formation from 6.4 on day 1 to 0 by day 7, indicating superior suppression of *A. baumannii* biofilm.

Similarly, in Fig. 8c, the GeL film had the least effect on *S. epidermidis*, reducing the biofilm count from 4.4 on day 1 to 0 by day 7. The Ag/ZnO 0.05@GeL film achieved moderate inhibition, decreasing biofilm levels from 5.4 to 0. The Ag/ZnO 0.1@GeL film demonstrated improved activity, lowering biofilm formation from 4.6 to 0. The Ag/ZnO 0.2@GeL film again exhibited the most potent antibiofilm effect, reducing *S. epidermidis* biofilm from 6.7 on day 1 to complete eradication by day 7.

Results depicted in Fig. 8d reveal the GeL film showed moderate formation of *S. pyogenes* biofilm, with a count of 3.6 at 1 day, decreasing to 0 at 7 days. The Ag/ZnO 0.05@GeL film displayed better biofilm reduction, starting at 4.4 at 1 day and dropping to 0 at 7 days. The Ag/ZnO 0.1@GeL film showed a similar trend, with biofilm counts starting at 3.5 at 1 day and reducing to 1.4 by 7 days. The Ag/ZnO 0.2@GeL film exhibited the highest biofilm suppression, with counts starting at 6.4 at 1 day and decreasing to 0 by 7 days.

The synthesized AgNPs functionalized with indole-3-acetic acid (IAA) exhibited remarkable antibiofilm activity, achieving 89% inhibition of biofilm formation. This substantial reduction highlights the potential of AgNPs-IAA in disrupting the initial stages of biofilm development, likely by interfering with bacterial adhesion and quorum sensing pathways^65^. The control of biofilm is a crucial consequence of Ag-doped ZnONPs’ antibacterial activity^66^. Biofilm, which are extracellular polymeric matrices produced by bacteria, protect bacterial colonies from external threats, including antibiotics and the host immune system^67,68^. However, Ag-doped ZnONPs have been shown to interact with EPS of biofilm^69,70^. The Ag-doped ZnONPs attach to the EPS matrix, disrupting its structure and facilitating the penetration of other antibacterial agents or the NPs themselves into the biofilm.

The antimicrobial activity of ZnONPs stands out among metal oxides due to both their excellent bactericidal effects and limited toxicity toward human cells^71^. Besides, ZnONPs display high antibacterial effects due to their ROS-producing capability that damages bacterial membranes to cause cell death^72^. ROS-generating systems demonstrate multiple benefits with their compatibility with living organisms and their ability to be readily reproduced while being eco-friendly and affordable. Based on recent research, scientists have found that the biological synthesis of ZnONPs with biofilm-like morphologies enhances these properties^73^.

Furthermore, the physical disturbances caused by the interaction between ZnONPs and AgNPs and the bacterial membrane are another key factor in the antibacterial activity. These interactions destabilize the membrane, resulting in ion leakage, especially of intracellular molecules like potassium and nucleotides, which disrupt cellular processes. Moreover, the ROS produced by ZnONPs and AgNPs contributes to oxidative damage to bacterial membranes and intracellular components, further enhancing their antibacterial activity^74^. These structural changes are often accompanied by cell wall disorders, which further compromise the integrity of the bacterial cell. The increased penetrability of the bacterial membrane due to nanoparticle interactions allows for more significant disruption and further increases the efficiency of the antibacterial action. As a result, the bacterial membrane becomes more susceptible to damage, leading to the eventual collapse of the bacterial cell^75^.

### Quantification of generated ROS level

**Fig. 9:**
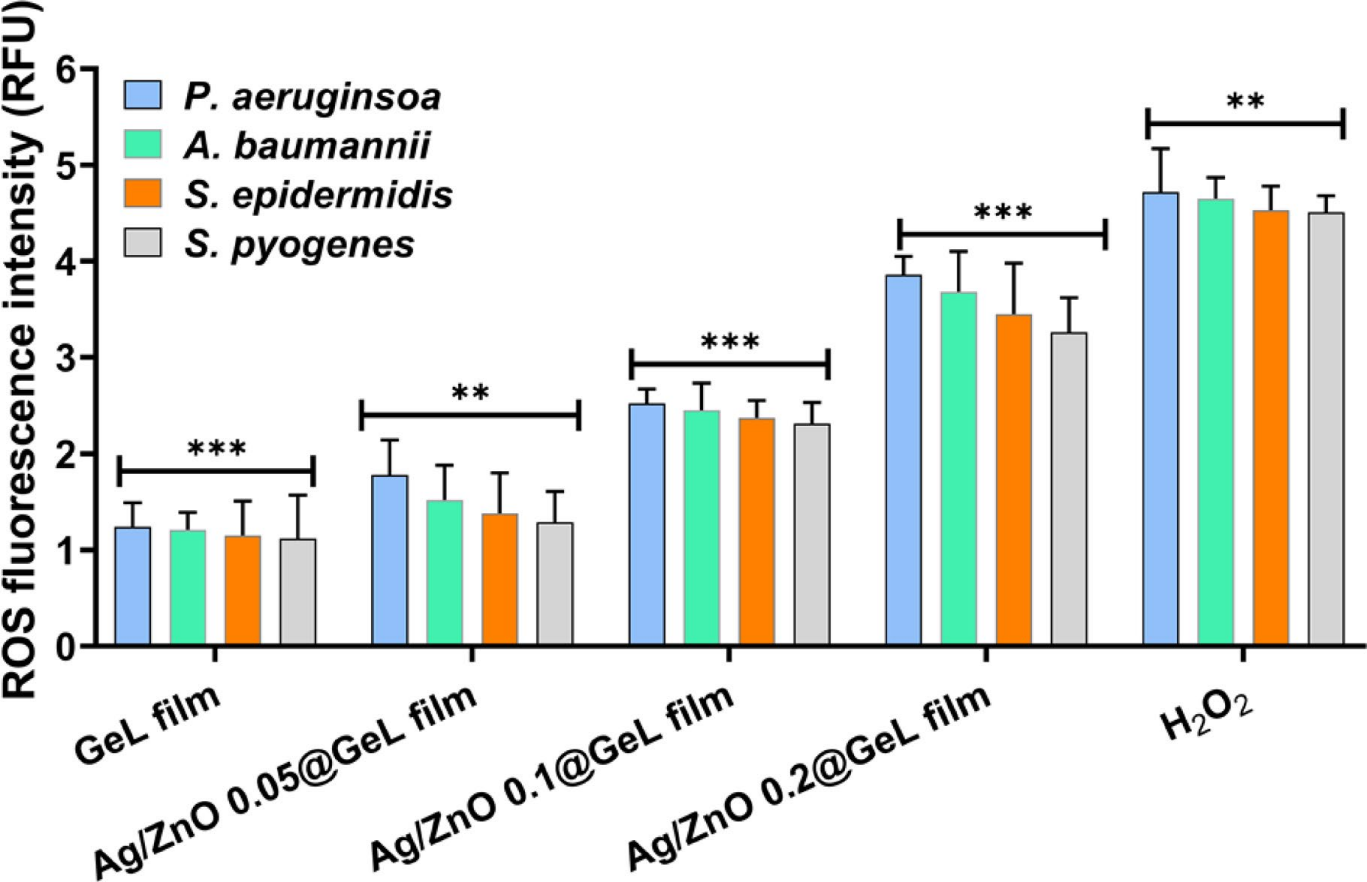
Intracellular ROS fluorescence intensity in four bacterial strains (*P. aeruginosa, A. baumannii, S. epidermidis,* and *S. pyogenes*) after treatment with GeL film, Ag/ZnO 0.05@GeL film, Ag/ZnO 0.1@GeL film, and Ag/ZnO 0.2@GeL film. ROS levels were measured using the DCFH-DA assay and are expressed in relative fluorescence units (RFU). Data represent mean ± SD (n = 3).

One of the major reasons for bacterial inactivation by disrupting the cell wall and cellular membrane is the generation of ROS when nanoparticles interact with bacterial cells. This happens when foreign particles interact with bacterial solution. Therefore, in this study, we examined the amount of ROS produced by each bacterial strain after exposure to the evaluated GeL-based films (GeL film, Ag/ZnO 0.05@GeL film, Ag/ZnO 0.1@GeL film, and Ag/ZnO 0.2@GeL film). Figure 9 illustrates the intracellular ROS levels in *P. aeruginosa, A. baumannii, S. epidermidis,* and *S. pyogenes* after treatment with GeL film, Ag/ZnO 0.05@GeL film, Ag/ZnO 0.1@GeL film, and Ag/ZnO 0.2@GeL film. Results reveal that among these selected bacterial strains, *P. aeruginosa* showed the highest ROS level when treated with Ag/ZnO 0.2@GeL, reaching 3.9 ± 0.15 RFU, compared to 1.2 ± 0.08 RFU in the GeL only control (*p* < 0.001). Similarly, ROS levels in *A. baumannii* increased from 1.2 ± 0.06 RFU (control) to 3.7 ± 0.12 RFU with Ag/ZnO 0.2@GeL (*p* < 0.001). In *S. epidermidis* and *S. pyogenes*, ROS levels increased from 1.1 ± 0.05 RFU to 3.5 ± 0.13 RFU and 1.0 ± 0.07 RFU to 3.3 ± 0.11 RFU, respectively, under the same treatment conditions. These values were statistically comparable to those observed with the positive control (H₂O₂), which ranged from 4.5 to 4.7 RFU, further supporting the role of Ag-doped ZnONPs in inducing oxidative stress. The results confirm that ROS generation, a major antibacterial mechanism in Ag/ZnO 0.2@GeL film, is the most effective in triggering oxidative damage across all tested strains.

The enhanced antibacterial activity of the Ag/ZnO@GeL films observed in this study strongly correlates with their ability to induce elevated levels of intracellular ROS in bacterial cells. As shown in Fig. 9, ROS levels increased in a dose-dependent manner across all tested strains, with the Ag/ZnO 0.2@GeL film formulation inducing the highest oxidative stress, closely matching the effect of the H₂O₂ positive control. This elevated ROS generation disrupts bacterial homeostasis by damaging cellular components such as DNA, proteins, and membrane lipids, ultimately leading to cell death^76^. The trend in ROS production closely mirrors the antibacterial performance of the films, where Ag/ZnO 0.2@GeL film consistently exhibited the most potent bactericidal effect, including complete inhibition of biofilm formation and high levels of membrane disruption. These findings support the hypothesis that ROS-mediated oxidative stress is a key mechanism underlying the antibacterial efficacy of Ag-doped ZnONPs. Moreover, the synergistic effect of AgNPs and ZnONPs (Ag-doped ZnONPs) in generating ROS enhances the bactericidal action without relying solely on traditional antibiotic mechanisms, making these films a promising candidate for combating multidrug-resistant pathogens in chronic wound environments.

### Antioxidant activities of Ag-doped ZnONPs loaded gelatin films

**Fig. 10:**
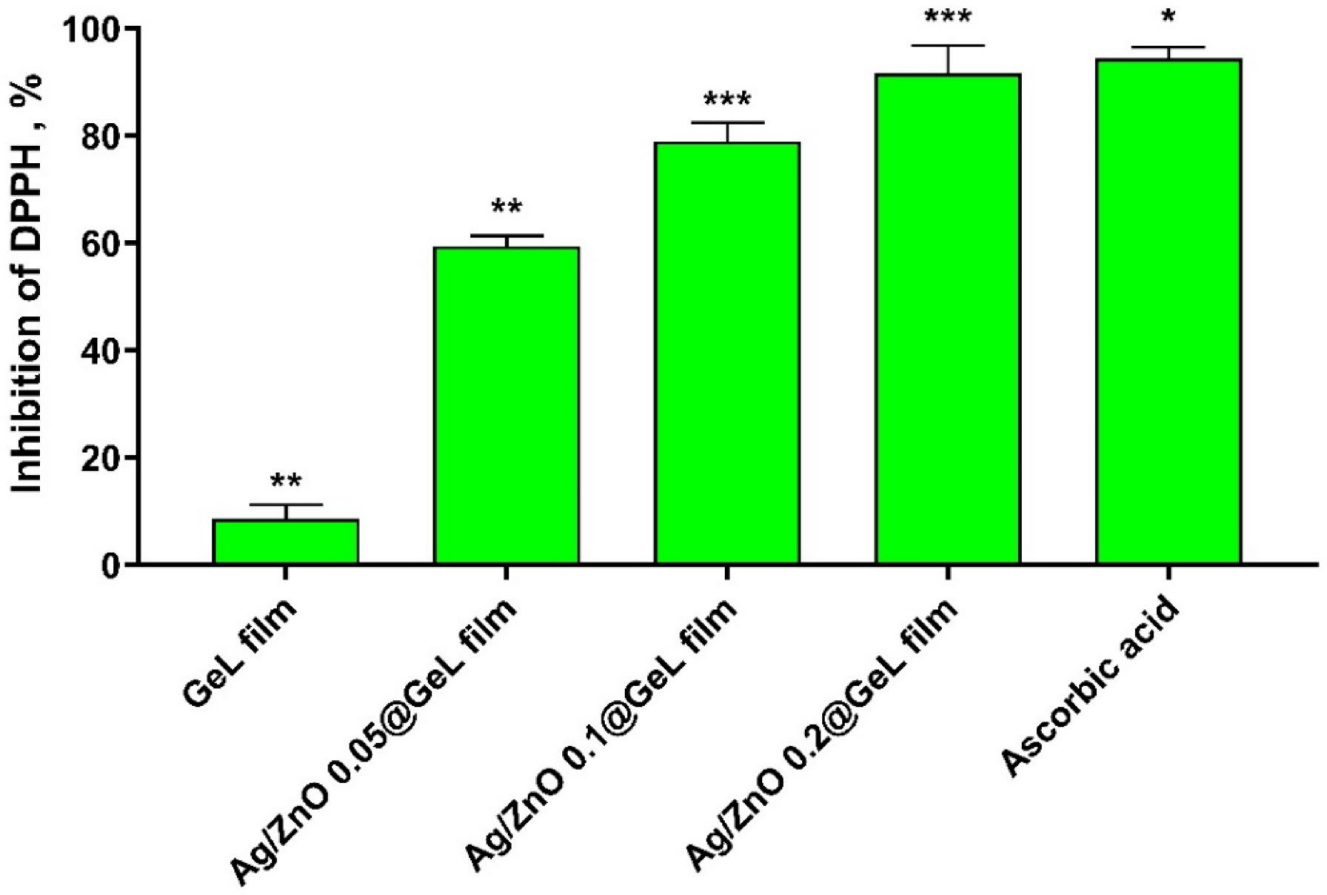
Percentage inhibition of DPPH radical scavenging activity by GeL film, Ag/ZnO 0.05@GeL film, Ag/ZnO 0.1@GeL film, and Ag/ZnO 0.2@GeL film compared to ascorbic acid as a positive control.

Figure 10 presents the DPPH radical scavenging activity of GeL film, Ag/ZnO 0.05@GeL film, Ag/ZnO 0.1@GeL film, and Ag/ZnO 0.2@GeLfilm, showing the percentage inhibition of DPPH scavenging radicals. The GeL film exhibited the lowest antioxidant activity, with 8.6% inhibition, indicating minimal scavenging of free radicals. The Ag/ZnO 0.05@GeL film demonstrated a moderate level of DPPH inhibition at 59.4%, suggesting that even a low concentration of Ag-doped ZnONPs enhances the antioxidant potential. The Ag/ZnO 0.1@GeL film further increased the inhibition to 78.9%, highlighting the progressive improvement in antioxidant activity as the concentration of Ag/ZnO increased. The Ag/ZnO 0.2@GeL film exhibited the highest scavenging activity, with 91.6% inhibition, nearly equivalent to ascorbic acid (94.4%), the positive control. These results indicate that the Ag/ZnO@GeL film, particularly those with higher concentrations of Ag-doped ZnONPs, possess significant antioxidant properties, which could be beneficial in wound healing applications by reducing oxidative stress and promoting tissue regeneration.

### Biocompatibility assessment of Ag-doped ZnONPs loaded gelatin films

**Fig. 11:**
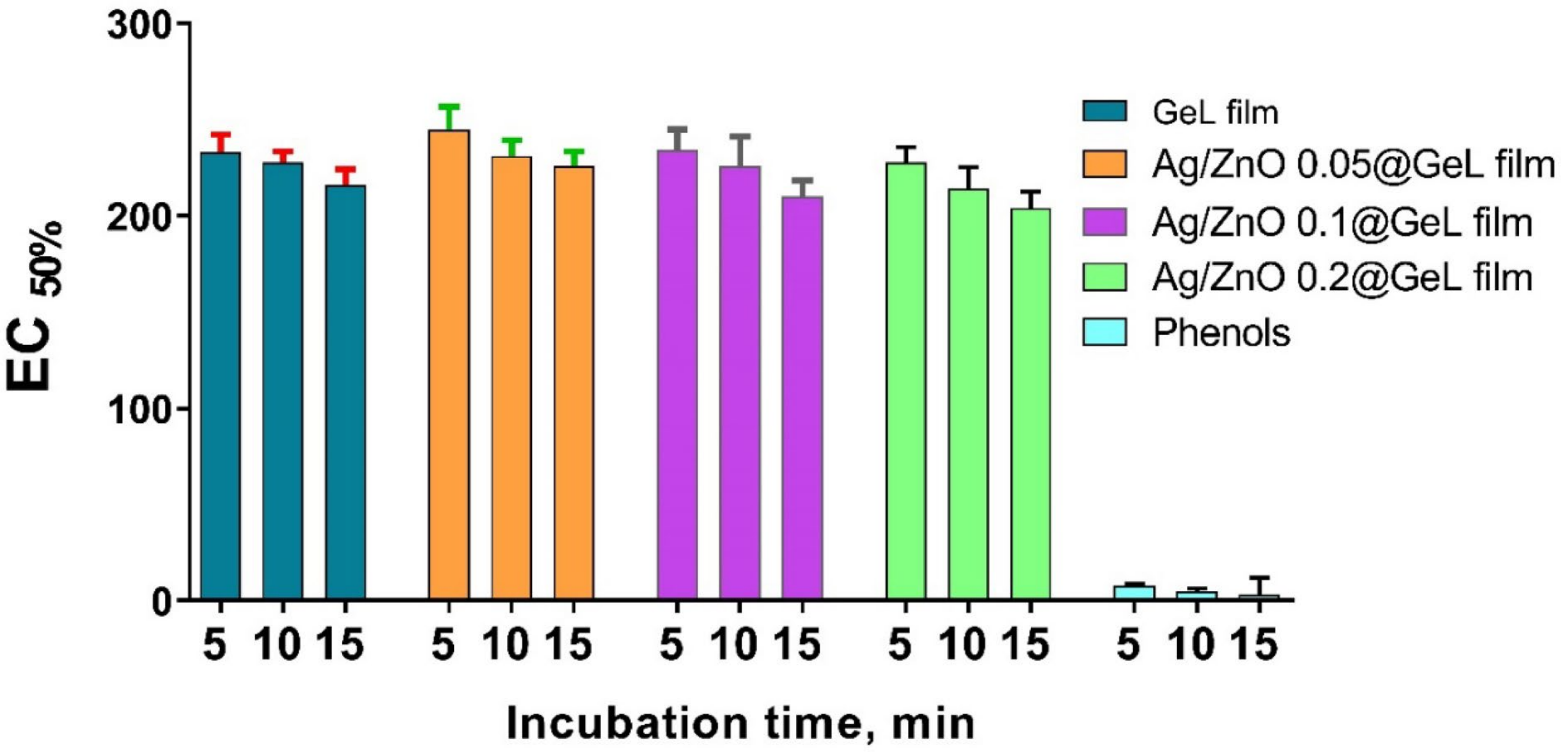
Toxicity assessment and EC_50%_ values of GeL film, Ag/ZnO 0.05@GeL film, Ag/ZnO 0.1@GeL film, and Ag/ZnO 0.2@GeL film) over different incubation times (5, 10, and 15 min).

The toxicity and biocompatibility of the tested film formulations (GeL film, Ag/ZnO 0.05@GeL film, Ag/ZnO 0.1@GeL film, and Ag/ZnO 0.2@GeL film) were evaluated using the Microtox analyzer, a well-established method to assess the safety of materials for biomedical applications. The results in Fig. 11 indicate that all the examined films demonstrated excellent safety profiles, with EC_50%_ values consistently exceeding 100 across all tested time points (5, 10, and 15 min). The EC_50%_ value is a critical indicator of toxicity, with a higher value signifying lower toxicity. The fact that all film formulations maintained EC_50%_ values above 100 at these time intervals confirms that the films are non-toxic and highly biocompatible, making them suitable for applications that require close contact with biological tissues, such as tissue engineering scaffolds and wound dressings. In contrast, the positive control phenol exhibited much lower EC_50%_ values (8, 5, and 3 at 5, 10, and 15 min, respectively), classifying it as highly toxic. This stark difference further supports the exceptional safety profile of the Ag/ZnO@GeL films. The excellent biocompatibility of these films suggests their potential for use in biomedical applications, where minimal adverse effects are crucial for promoting healing and minimizing risks to the patient. These findings underscore the promise of the Ag/ZnO@GeL films as safe materials for therapeutic and wound healing purposes. This study emphasizes the exceptional biocompatibility of ZnONPs, demonstrating their suitability for a wide range of biomedical applications. However, it is important to carefully assess the potential cytotoxicity of Ag-doped ZnONPs on human cells^77^. While the inclusion of AgNPs significantly enhances the antibacterial properties of the Ag-doped ZnONPs, it also introduces concerns regarding potential toxicity at higher concentrations^39^.

## Conclusion

This current study reported the preparation of gelatin-based films loaded with different concentrations of Ag-doped ZnONPs and assessing the structural, physiochemical, and antibacterial properties of the obtained films to evaluate the synergistic effect of Ag-doped ZnONPs loaded gelatin (GeL) film as a promising antibacterial wound dressing material. SEM images showed that Ag-ZnO@GeL films had a coarse structure compared to the pristine GeL film with uniform distribution of Ag-doped ZnONPs. The incorporation of Ag-doped ZnONPs significantly enhanced the antibacterial, antibiofilm, antioxidant, and anticancer properties of the GeL films, with the Ag/ZnO 0.2@GeL film demonstrating the most potent and broad-spectrum activity. The films exhibited strong antibacterial effects against both Gram-positive and Gram-negative pathogens, with complete inhibition of biofilm formation in several strains. ROS quantification assays confirmed that the antibacterial mechanism is closely linked to oxidative stress induced in bacterial cells. In vitro biocompatibility assays usong Microtox verified that the optimized films maintain no toxicity profiles while achieving high efficacy. This study provides a promising platform for developing next-generation antimicrobial wound dressings, particularly relevant in the context of rising antibiotic resistance and chronic wound management. Further, the findings suggest that Ag/ZnO@GeL films offer a promising approach for wound healing applications, providing antibacterial and biocompatible properties.

## Data Availability

The datasets used and/or analyzed during the current study are available from the corresponding author upon reasonable request.
